# Educational differentials in the realisation of fertility intentions: Is sub-Saharan Africa different?

**DOI:** 10.1371/journal.pone.0219736

**Published:** 2019-07-18

**Authors:** Melanie Dawn Channon, Sarah Harper

**Affiliations:** 1 Department of Social and Policy Sciences, University of Bath, Bath, United Kingdom; 2 Oxford Institute of Population Ageing, University of Oxford, Oxford, United Kingdom; USC Keck School of Medicine, Institute for Global Health, UNITED STATES

## Abstract

**Background:**

The gap between fertility outcomes and fertility ideals is notably higher in sub-Saharan Africa (SSA) than elsewhere, relating to both under- and overachievement of fertility ideals. We consider the extent to which the relationship between fertility ideals and fertility outcomes is related to educational achievement. Further, we consider if these educational differentials are the same or different in SSA, and thereby consider the extent to which increasing levels of education in SSA may decrease fertility.

**Data and methods:**

We use 227 Demographic and Health Surveys (DHSs) from 58 countries worldwide to look at population- level measures of the mismatch between fertility ideals and fertility outcomes. Population level measures are used to assess whether the correspondence between fertility intentions and achievements differ by level of education. We then look at the individual-level determinants of both under- and overachieving fertility intentions. Data from the most recent DHS in 54 of the original countries is used for the individual level analysis, with five countries excluded due to the most recent available survey being out of date.

**Results:**

An average of 40% of women in SSA underachieve their stated fertility intentions compared to 26% in non-SSA countries. Furthermore, compared to other LMICs, higher levels of education are not related to better correspondence between fertility intentions and outcomes in SSA. In Middle/Western Africa countries, on average, 48% of women with secondary or higher education have fewer children than their ideal, compared to just 24% who have more children than their ideal.

**Conclusion:**

We argue that the phenomenon of underachieving fertility ideals (or unrealized fertility) may be of particular importance for the ongoing fertility transition throughout SSA, especially as more highly educated groups do not appear to be following the patterns observed elsewhere.

## Introduction

The misalignment of childbearing goals and childbearing outcomes has long been seen as an important area of research, but in low and middle income countries (LMICs) this research has predominantly focussed on unintended fertility i.e. women having more children than they would like [11]. The other misalignment is when fewer children are borne than are wanted and this has received relatively little attention in the context of higher fertility, a point previously discussed by the authors of this paper [2–4] and recently highlighted as an important area of enquiry for the future of demography by John Casterline at the Population Association of America Presidential Address [5]. This paper looks at both misalignments in combination, as these are two sides of the same issue; looking at one without the other ignores the complex reality of childbearing choices.

Previous research has found that the mismatch between fertility outcomes and fertility ideals at an aggregate level is notably higher in sub-Saharan African (SSA) countries as compared to other LMICs [6]. In particular, the mismatch between fertility ideals and outcomes does not decrease with education as might be expected, but rather fertility ideals continue to exceed reality even with increased education. The concept of continuing to underachieve fertility ideals despite increased education has been seriously neglected in the context of LMICs, and particularly in the SSA context [1, 4, 5, 7, 8]. It might be that SSA countries appear exceptional simply due to the much lower levels of education. A priori we might generally expect women with lower levels of education to have higher levels of unwanted births when compared to more highly educated women, and find it more difficult to translate their fertility ideals or intentions into reality.

In sub-Saharan Africa fertility ideals (the number of children people say they want) are particularly high, as has been noted in previous research [1, 9]. Furthermore, it has widely been argued that fertility transition in Africa differs from that of other regions [10, 11]. It has been seen to be distinctive firstly because of its pronatalism [9], and secondly because of reductions in the overall fertility rate resulting from the lengthening of birth intervals at all parities rather than stopping behaviour [12]. The mean desired family size in sub-Saharan Africa is 5.0, as compared to 2.9 in other LMICs (authors’ own calculations), while previous research has highlighted the need to understand the sustained high demand for children particularly in Middle and Western Africa [11]. Given that sub-Saharan Africa is the world’s fastest growing region, with a population projected to more than double by 2050, it is essential to understand the drivers of the relationship between fertility ideals and outcomes. This paper seeks to study if the relationship between fertility ideals and fertility outcomes (at both the aggregate and individual level) varies by geographical region and educational level and if these two factors interact with each other.

This paper considers educational differentials in the extent to which fertility ideals are successfully translated into fertility outcomes. Specifically we compare responses to the question “If you could go back to the time you did not have any children and could choose exactly the number of children to have in your whole life, how many would that be?” and responses to questions about actual number of children ever born.

One common argument is that increasing education will reduce desired fertility, thus reducing fertility. Educational differentials in fertility have long been a feature of demographic research, with Caldwell arguing that mass education was the primary driving force behind fertility transitions [13]. A number of mechanisms might contribute towards the relationship between education and fertility including increased autonomy, exposure to new childbearing and gender norms, increased employment opportunities and thus opportunity cost of childbearing, improved health behaviours, and the indirect effects of delayed marriage and decreasing child mortality [14–18]. Of course, we must also consider that observed educational differences in fertility might be the result of selectivity rather than the direct effects of increased educational attainment and time spent in schooling. This endogeneity has been addressed in some previous research, with one study looking particularly at desired family size in Malawi, Uganda and Ethiopia finding that education still has a significant effect when endogeneity is accounted for, though that effect is attenuated [17]. Another important point about education is that quantity alone may not be sufficient, when the quality is low, especially following large recent expansions of education in many low and middle income countries [19].

In sub-Saharan Africa while both fertility outcomes and desired family size fall with increasing education, achieved fertility appears to fall much more substantially than desired fertility. Even relatively highly educated women have high desired fertility in much of sub-Saharan Africa (a mean of 3.7 for women with higher education, versus 2.7 for women in other LMICs) (authors own calculations). Despite their high desired family size more highly educated women do have fewer children than their less educated counterparts [10]. In fact, more educated women often fall short of their stated ideal number of children with desired family size exceeding the TFR by roughly one whole child in sub-Saharan Africa for those with higher education, compared to half a child for other LMICs [3].

What does ideal family size mean if more educated women consistently have fewer (often far fewer) children than they say is ideal? Previous research in four African countries found that neither education nor standard women’s empowerment indicators show a strong or consistent relationship with achievement of fertility ideals [20]. However, this research concentrated only on overachieving fertility ideals, rather than considering the further difference between women who have exactly their ideal number of children and those who underachieve. To add to the complexity it is important to consider the extent to which fertility ideals observed at a single point in time reflect the reality of fertility preferences. Recent research has argued that fertility preferences are flexible in their nature, especially in the context of uncertainty about future prospects and stability as is the case in many low and middle income countries [21].

The concept of underachieving fertility ideals has been seriously neglected in the context of low and middle income countries, while the literature on this phenomenon within high income countries is relatively well developed [22–25]. To date we could find just one published analysis, written by Casterline and Han, that provides a comparative focus on low and middle income countries [7]; this paper provides an important first overview of underachieved fertility (though the authors use the term unrealized fertility) in 78 low and middle income countries, with the inclusion of some important demographic covariates. Casterline and Han do not look at the relationship between underachieving fertility ideals and education or look at this in combination with unplanned fertility, which is a significant next step.

Our paper argues that the phenomenon of underachieving fertility ideals is a potentially important, but neglected, aspect of future fertility decline in sub-Saharan Africa. The gap between ideal and actual fertility is important in that it might signify the potential for future fertility decline or the potential for a future stall—or even rebound—in fertility. The particular tendency for highly educated women in sub-Saharan Africa to underachieve fertility intentions, given that we would expect such women to be empowered to achieve their reproductive ideals, is puzzling. It has implications for future research concerning the nature of the data that we are collecting on fertility ideals as well as well as how we choose to analyse and interpret that data.

### Research questions

1. Does the relationship between fertility ideals and fertility outcomes vary by educational level?
  1.1 Does any educational variation differ across regional LMIC groupings?
2. Are changes in fertility ideals driven more by increasing levels of education, or changes in fertility ideals within educational groups?
  2.1 Does this differ across regional LMIC groupings?
3. What is the relationship between education and underachieving, equalling, and overachieving fertility ideals?
  3.1 Does this relationship differ across regional LMIC groupings?

## Materials and methods

Demographic and Health Surveys (DHSs) were used from every country with available data, with a small number of exclusions. This amounted to 227 surveys across 58 countries. For descriptive analyses we split the countries into four regions: western/middle Africa, eastern/southern Africa, Asia and North Africa, and Latin America and the Caribbean. For individual level analyses, we only used the most recently available DHS for each country in order to provide an overview of the current state of achieved fertility compared to fertility ideals (as noted in S1 Table). In order to ensure that a contemporary overview we excluded surveys conducted prior to round 4 of the DHS, meaning that all surveys used in the individual level analyses took place between 2003 and 2015. This resulted in the exclusion of five countries that were used in aggregate level analyses: Brazil, Eritrea, Guatemala, Kazakhstan, and Vietnam.

The simplest and most intuitive measure of fertility desires is ideal family size, or the average ideal number of children (AINC). This measure uses the answers provided to the following question: “If you could go back to the time you did not have any children and could choose exactly the number of children to have in your whole life, how many would that be?” This has some important limitations. Firstly, non-numeric responses (such as “up to God”) are problematic, though the make up less than 10% of the responses in the majority of surveys. Non-numeric responses have been found to be much more common amongst those with less education and a lower level of contraceptive knowledge, though such responses are increasingly rare [26]. We excluded non-numeric responses (in line with Casterline and Han) as they could not be incorporated into the AINC and such responses suggested that the very idea of a mismatch between fertility ideals and outcomes would make no sense to respondents who answered in this way [7]. A second limitation is that respondents are less likely to respond with an ideal number of children that is less than their current number of children. In other words, respondents may have prospectively wanted no more children, but if a further child is born they will upwardly adjust their ideal number of children. This relates to a third problem, which is that the ideal number of children may not be constant throughout the life course. The underlying ideal number may vary as childbearing decisions are often taken sequentially by parity. Recent research has also described how fertility preferences in Africa may be “strategically flexible” in response to life events (e.g. death, divorce, HIV infection and many others)[21, 27]. A further point is that the primary fertility goal may not be a number of births (which is generally how the AINC is interpreted). The primary fertility goal may be a specific number of children net of any childhood deaths, a specific number of boys or girls, or a balanced number of boys and girls. Alternatively, the ideal number of children may simply be unattainable due to outside constraints such as infertility or availability of a partner.

Previous research has tended to use constructs such as called the wanted TFR (WTFR), the desired TFR (DTFR) and the wanted fertility rate (wTFR) to consider the effect of fertility ideals on actual fertility [6, 28–31]. For our purposes these measures are not sufficient as none of them can incorporate the idea of underachieving fertility ideals. We recognise that the ideal family size question incorporates many aforementioned biases, but in the absence of longitudinal data it is the best measure available. Furthermore, it has the advantage of being freely available across DHSs, which collect data in the majority of LMICs and, in many countries, do so frequently.

At an individual level the ideal number of children can be compared to the number of children ever born to establish if an individual has overachieved, underachieved or equalled their fertility ideals. Of course, it is only possible to do this after childbearing is completed. In line with previous research we restrict our sample to women aged over 40 for individual level analyses. It would also be possible to use the question about intending to have another child to consider whether a woman had underachieved her fertility ideals. The relevant question within the DHS is: “Now I have some questions about the future. Would you like to have (a/another) child, or would you prefer not to have any (more) children?” A possible response to this is for the woman to say that she can’t get pregnant, which would be the likely response for a woman aged 40 or over. This is not to say that her ideal number of children has been achieved. Thus, this question, which has frequently been analysed, is not as useful for analysing achievement of fertility ideals amongst those who have completed childbearing.

In terms of education, for the majority of analyses we split education into four levels: none, primary, secondary and higher. However, for some surveys the sample within these educational groupings is very small. This is especially the case for older women in some sub-Saharan African countries, where it has been necessary to collapse the secondary and higher education categories. For the country level analysis the variables (TFR and AINC) were calculated separately for each educational level. For the individual level analysis we took the decision to combine secondary and higher level education to reduce the number of countries removed from our analysis due to small sample sizes. In addition we exclude European countries that were surveyed (e.g. Albania, Moldova etc.) because the levels of education are so high that a comparison is not possible for less educated individuals.

We conducted a decomposition analysis to study the effects of education on overall changes in desired family size. Following the method used by Weinberger et al. [32], the overall change is split into two components: the composition effect and the rate effect. The composition effect captures the impact of changing educational attainment, while the rate effect captures the impact of changes in education-specific desires.

We use four geographical regions within our analysis: Western/Middle Africa, Eastern/Southern Africa, Asia/Northern Africa and the Middle East, and Latin America and the Caribbean. Initially we conducted analyses with sub-Saharan Africa as one geographical region, but a sensitivity analysis indicated that there were substantial differences between Western/Middle and Eastern/Southern Africa, which is in line with previous research [33].

We conducted a series of two-way 4 (region) x 3 (level of education) mixed ANOVAs with repeated measures within country for the different levels of education; a separate ANOVA was conducted to look at the percentage of women who overachieved fertility ideals, the percentage who equalled fertility ideals, and the percentage who underachieved fertility ideals. We used these to test whether there were significant differences between geographic regions, and education levels, and to test whether there was a significant interaction between these two factors.

There are several limitations to our analysis. Firstly, we were limited in the countries that we could include in our analysis as DHSs were not conducted in every country at every time point. Furthermore, the countries for which we have data may not be representative of the region as a whole. Coverage is particularly low in Latin America (e.g. Argentina and Mexico are missing). Some of the surveys included are not recent (e.g. the latest survey in Kazakhstan was conducted in 1999). In some surveys only women who are currently in a union are asked about their fertility preferences. Perhaps most importantly, though, aggregate analyses mask country level trends that may be substantially different to overall and regional trends. Information on the countries and survey years included in the analyses are provided in the supplementary material (S1 Table).

## Results

**Fig 1:**
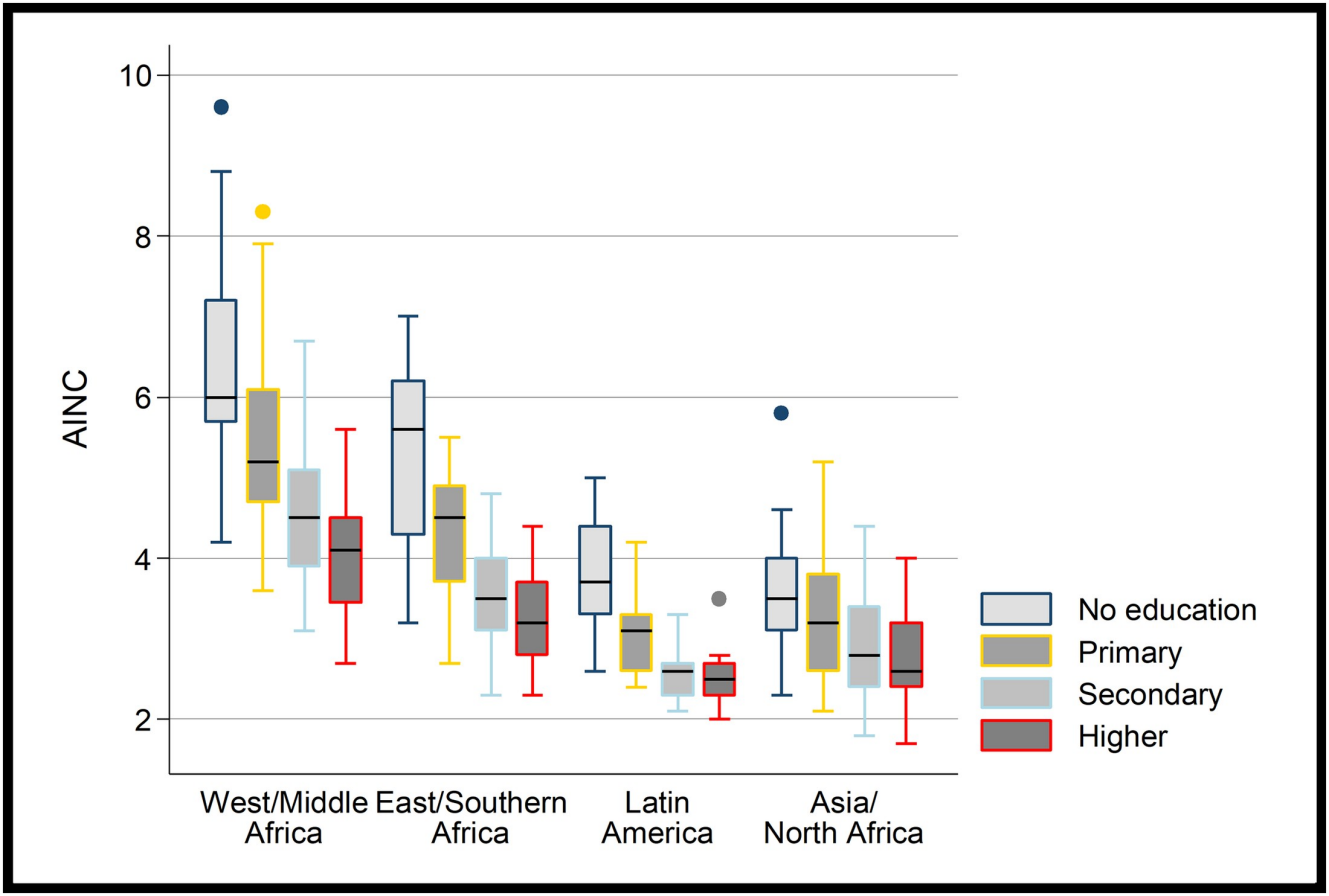
Average ideal number of children (AINC) by education and region.

Desired family size (as measured by the AINC) is higher in sub-Saharan Africa for women of every educational level, but this is particularly the case in Western/Middle Africa compared to Eastern/Southern Africa (see Fig 1). For women with no education the mean AINC is 5.9 in SSA countries compared to 3.6 elsewhere—a difference of 2.4 children. For women with higher education the difference between SSA countries and elsewhere is smaller, but the mean AINC in SSA is still a whole child more than other observed countries, at 3.7. A small group of African countries (Central African Republic, Chad and Niger) stand out as having an AINC above 5.0 for women with higher education; however, it should be noted that the most recent survey for the Central African Republic was conducted in 1994–95 so this dataset is from a much earlier time period to that from most of the other countries. This is an extraordinarily high number for such a high level of education. However, the actual level of fertility for the highly educated women in these three countries is relatively low (the TFR ranges from 1.9 to 3.6) when compared to the overall level (which ranges from 5.1 to 8.0). There is no country in Africa for which the AINC is at replacement level or below, even for the most highly educated. The lowest AINC was observed in Swaziland at 2.5, with Lesotho at 2.6; for women with higher education in these countries the AINC was 2.3 and 2.5 respectively. No African country outside of Southern Africa was observed to have a desired family size below 3.0 and most had desired family sizes above 4.0.

**Fig 2:**
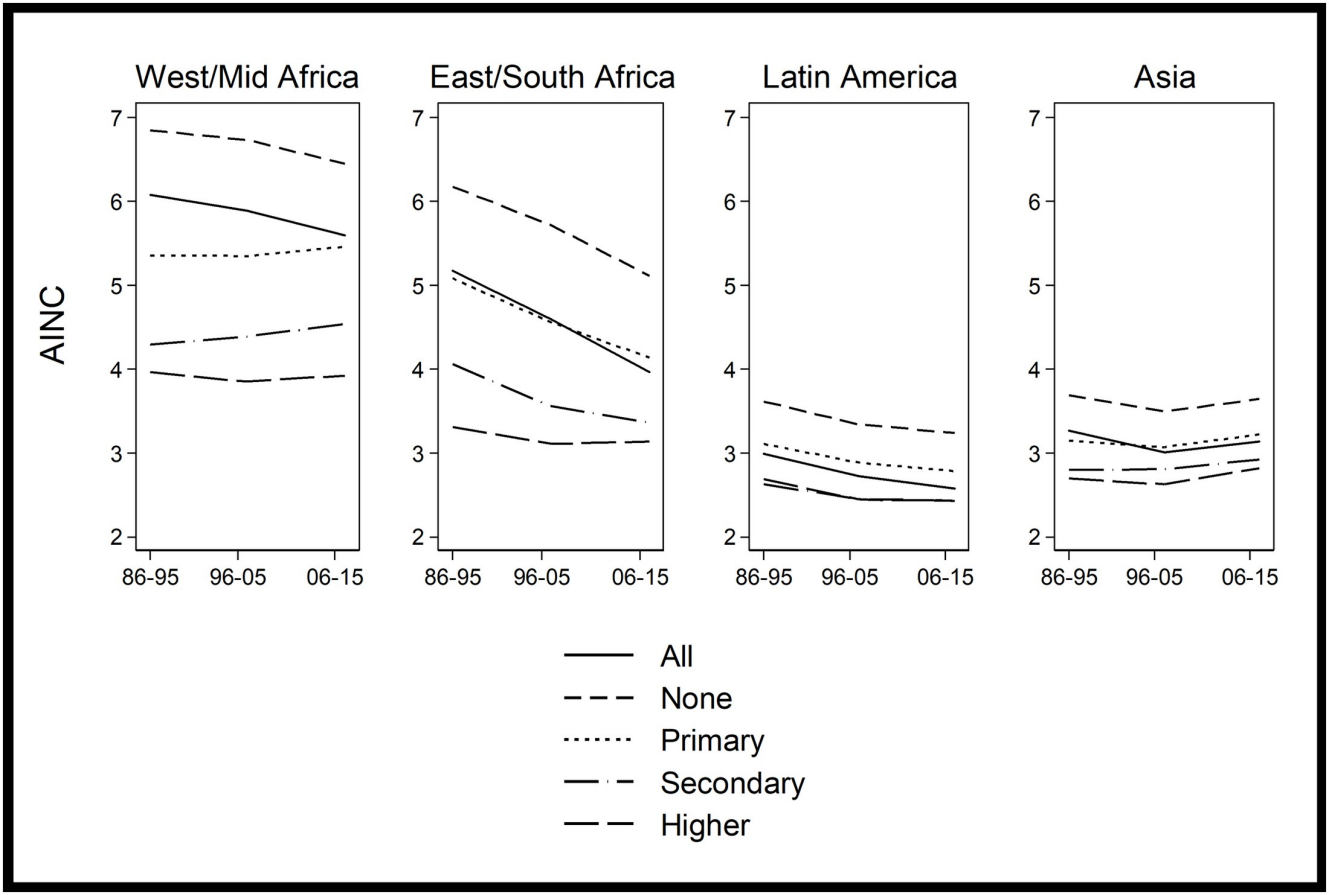
Trends in average ideal number of children by region and educational level.

One explanation for the high desired family sizes observed amongst highly educated women in sub-Saharan Africa is that these countries have much higher levels of overall fertility than most other LMICs. At earlier stages of the fertility transition perhaps other countries exhibited a similar pattern. Looking at earlier DHSs in non-African countries we find that the AINC for women with higher education was still very low (Fig 2). For countries with a TFR greater than 4.0 the mean AINC in non-African countries was 2.9 for women with higher education and 3.0 for women with secondary education, compared to 3.6 and 4.1 for sub-Saharan African countries.

**Fig 3:**
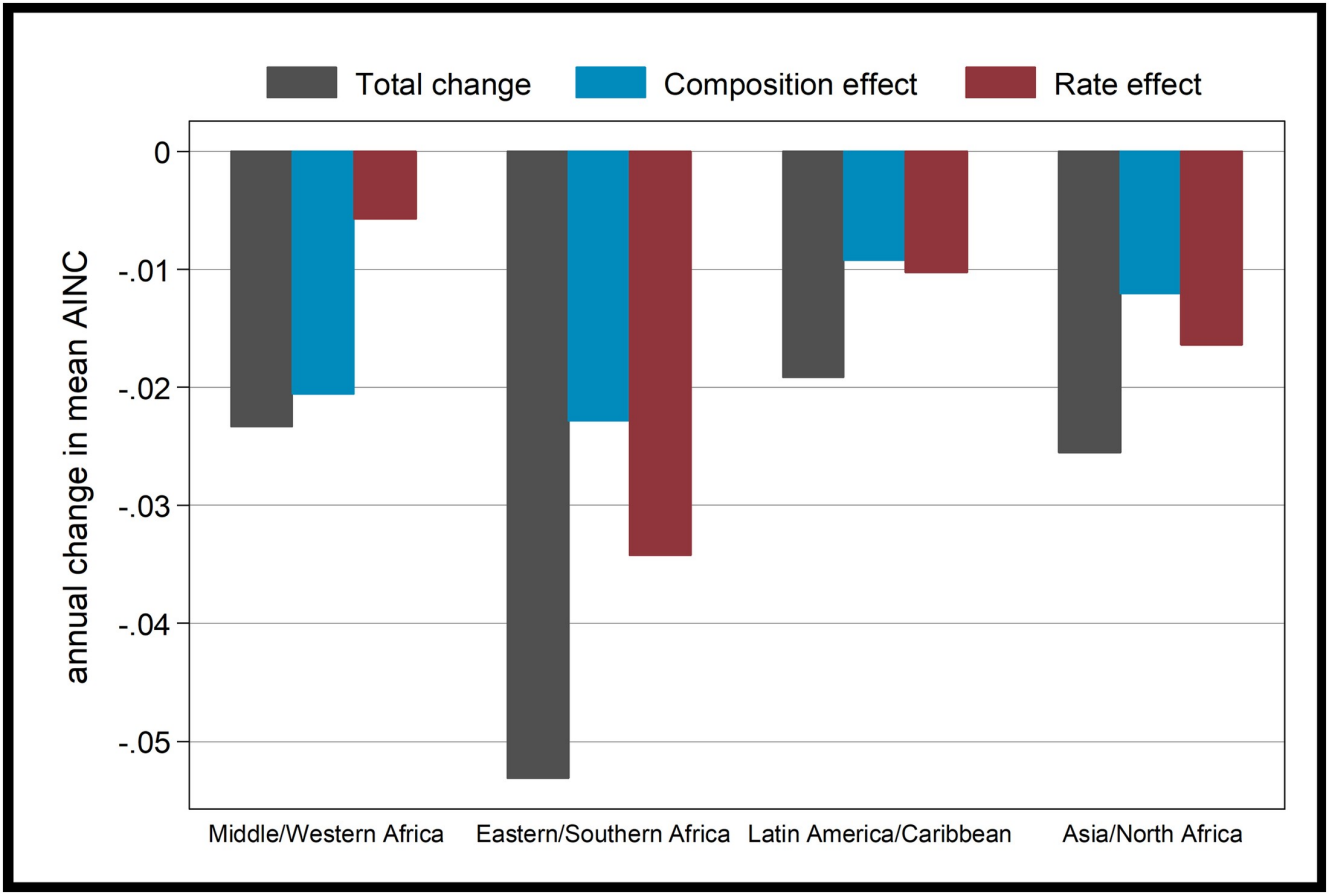
Annual change in the average ideal number of children in each region, decomposed.

Results of the decomposition analysis are shown in Fig 3 for each region separately. This shows that the largest observed total annual change was in Eastern/Southern Africa, where the rate effect was also particularly high, indicating that substantial changes to desired family size were observed within educational groups (as can also clearly be seen in Fig 2). Interestingly, the rate effect dominates the composition effect in all regions apart from Middle/Western Africa, where the rate effect is extremely small. In the case of Middle/Western Africa the rate of annual change in AINC is not unusual, but the fact that this change is almost entirely a result of changing educational composition is unusual. In Fig 2 it is clear that for most educational levels the AINC is not declining in Western/Middle Africa, although there is a modest decline amongst those with no education. Of course the educational composition of these regions varies dramatically, as do the fertility levels. Regions further along the fertility transition show a convergence of desired family size between different educational levels. It might be the case that once fertility begins to fall more substantially in Western/Middle Africa, desired family sizes at different levels of education will both fall and converge, but this is only a possibility and by no means certain.

The data show that African countries are not homogenous and that Middle/Western Africa is particularly pronatalist at all levels of education, even when compared to other countries with similar fertility levels. However, we have already noted that the fertility levels of more highly educated women in sub-Saharan Africa are not as high as the AINC would suggest.

So far we have studied only macro patterns, which is in line with previous work looking at the mismatch between fertility ideals and achievements. However, this misses the important issue of individuals’ ability to translate their fertility preferences into reality. We would expect, a priori, that more highly educated individuals would be better equipped to translate their preferences correctly. The country level mean of overachieving, underachieving and equalling fertility preferences is summarised in Table 1 for each of the four regions, both overall and by level of women’s education. We also provide individual country results S2 Table.

**Table 1:** Mean country level of overachieving, underachieving and equalling fertility desires by region and educational achievement.

		Overall	No education	Primary	Secondary/ Higher
**Underachieving**	Western/Middle Africa	45.0	43.9	42.9	48.4
	Eastern/Southern Africa	33.6	33.6	31.0	40.4
	Latin America/Caribbean	29.3	19.5	23.5	34.1
	Asia/Northern Africa	24.3	24.0	19.0	25.4
**Overachieving**	Western/Middle Africa	28.7	29.3	31.3	24.3
	Eastern/Southern Africa	39.8	40.0	43.0	30.7
	Latin America/Caribbean	41.9	56.9	50.5	32.9
	Asia/Northern Africa	38.9	43.2	46.1	34.8
**Equalling**	Western/Middle Africa	26.3	26.8	25.8	27.4
	Eastern/Southern Africa	26.6	26.5	26.0	28.9
	Latin America/Caribbean	28.8	23.6	26.0	32.9
	Asia/Northern Africa	36.8	32.8	34.9	39.8

From Table 1 we can see that, in the majority of surveys the proportion of women who reported that their ideal number of children equalled their achieved fertility was low. In Latin America and Asia/North Africa there was generally a positive educational gradient, with the most educated women being most likely to report achieving their ideal number of children (33% in Latin America/Caribbean and 40% in Asia/North Africa). On average in Latin America 40% more women with secondary or higher education reported equalling fertility desires than women with no education, while in Asia/Northern Africa the figure was 21%. In West/Middle and Eastern/Southern African countries, on the other hand, there was no visible difference between educational levels, with little more than a quarter of women reporting that they had achieved their ideal family size irrespective of their educational background. The results of the two way mixed ANOVA found that while the main effect of region was not significant, individual contrasts indicated that Asia/North Africa did have significantly different percentages of women equalling fertility desires compared to the other regions (p = 0.019).

The educational gradient seen in most countries (especially in sub-Saharan Africa) is weak or inconsistent, which is at odds with what we would expect to see. That said, the ANOVA analysis indicated that the main effect of education was significant (p<0.001), while the interaction between education and region was not. However, the individual contrasts indicated that there was no significant difference between no education and primary education, but there was a significant difference between no education and secondary/higher education (p = 0.001). Overall, it is clear that a mismatch between fertility ideals and outcomes (including both under- and overachievers) was common across all regions and educational levels, with the highest observed mean regional proportion of women saying that their fertility outcomes equalled their ideals at only 40% for those with secondary or higher education in Asia/North Africa (Table 1).

It is notable that the proportion of underachievers is particularly high amongst all educational levels in Western/Middle Africa, with 48% of those with secondary or higher education underachieving compared to 24% overachieving (Table 1). In Eastern/Southern Africa 40% underachieve and 31% overachieve. Whereas in non-SSA countries 28% underachieve and 34% overachieve, despite the fact that fertility is, on average, much higher in SSA countries. In other words, the most common outcome for women over 40 in Western/Middle Africa is that they report wanting more children than they actually have. Again, it is notable that there are few educational differences in terms of underachieving of fertility ideals, especially in sub-Saharan Africa.

We conducted further two way mixed ANOVAs to compare the main effects of education and region and the interaction between education and region on the percentage of women who overachieved and underachieved their fertility ideals. In both cases the main effect of education was found to be highly significant (p<0.001). The individual contrasts in both cases indicated that there was no significant difference between no education and primary education, but there was a significant difference between no education and secondary/higher education (p<0.001). The main effect of region was also highly significant for both overachieving (p = 0.004) and underachieving (p<0.001), making it clear that a woman’s ability to overachieve or underachieve her fertility ideals is related to which region she lives in. For overachieving the interaction between education and region was also significant (p = 0.039). This provides some evidence that education does have a differential effect depending on region, which is over and above the additive effects of education and region.

## Discussion

The lack of research in underachieving of fertility ideals (or unrealized fertility) in low and middle income countries within the demographic literature is surprising, especially when this theme can clearly be seen within the literature of other subjects such as anthropology and sociology [7].

There is large variation visible in sub-Saharan Africa both between regions and between individual countries, with particularly high desired fertility seen in countries in the Western/Middle region. Western/Middle Africa is particularly distinctive in terms of the large proportion of women who say that their ideal fertility is higher than their achieved fertility, that is underachievers; it is also distinctive for the extremely high desired family sizes (above four children) visible even amongst those with higher education, and the fact that education-specific desired family sizes have not shown substantial decline. One possible explanation is that the quality of schooling in many places within sub-Saharan Africa is relatively low and highly variable, and thus the effect it has on fertility desires may be lower than elsewhere [19, 33, 34]. Furthermore, the limited supply of high quality education will be particularly selective in terms of who has access to this. While increasing education in the region will likely lead to some reduction in desired family size, this reduction is unlikely to be as large as declines seen in other areas of the world without schooling expansion being accompanied by a focus on quality [19, 33].

Women underachieving their fertility ideals is common amongst women of all educational groups in sub-Saharan Africa. When highly educated women are not achieving their reported fertility desires due to underachieving, then that is an interesting scenario. These results indicate, first, that fertility has been declining faster than fertility preferences, in particular for those with higher education. This result is in line with Lam’s [35] finding that only 50% of fertility declines are explained by changes in fertility preferences and with Feyisetan and Casterline’s [36] finding that only 30% of the increase in contraceptive use can be explained by changes in fertility preferences. Fertility desires are far from the only driver of realised fertility, and it is clear that more educated women in many African countries have limited their childbearing substantially, despite sustained high fertility desires in many cases. One potential explanation for this is that more highly educated women are simply delaying childbearing and extending birth intervals at each parity without a specific desire to limit the number of children that they have [12, 37]. This is not dissimilar to patterns observed in more developed countries such as the UK, in that underachieving fertility desires is a common outcome for more highly educated women in these countries partly as a result of postponement [38, 39].

Second, in sub-Saharan Africa, while there is considerable scope for family planning programs (or other policies) to decrease unwanted births and to improve the transformation of fertility preferences into fertility outcomes for women with low levels of education, this is not the case for more highly educated women. Indeed, one might ask the question as to why more educated women in sub-Saharan Africa have relatively few children when they still report a desire for relatively large family sizes; it seems that there may even be an unmet need for children amongst more educated women in many sub-Saharan African countries. This is something which is yet to be well explored in the demographic literature. While high fertility desires are most certainly an obstacle to fertility decline, education appears to be associated with a reduction in fertility without the concomitant reduction in desires. All this lends further weight to the argument of African exceptionalism in the fertility transition. That said, within the demographic literature there are two broad explanations of why women might reach the end of their reproductive lifecourse without achieving their fertility ideals: the first of these is that women have competing life goals and it is not possible to pursue all those goals simultaneously (e.g. having many children and being in full time employment). The other broad explanation is that women “leave it too late”, which could be the result of many things including pursuing other life goals such as education. The delay in starting childbearing resulting from more education has been extensively discussed in the Western context, along with the implications for rising numbers of women who find that they have “left it too late” (see e.g. [40]), but the implications of such childbearing delays for more educated women in LMICs have not been well researched. It is generally taken that such a delay occurring in a context of high fertility is a universally good thing, when the high numbers of women who have underachieved their fertility intentions at the end of their reproductive lifecourse indicates that the reality may be much more complex.

An important methodological point to make is that the idea that women employing ex post rationalisation to report wanting the same number of births that they actually have is assumed to upwardly bias the AINC. However, given the large proportion of women who report wanting more children than they had, this bias may not have such a substantial effect as previously thought. Relatively small numbers of women report a desired family size that equals their actual family size. It may also be the case that the extent of such ex post rationalisations vary substantially depending on the cultural context as is the case with non-numeric responses [26].

Another possibility is that the high levels of underachieving fertility ideals in sub-Saharan Africa have a more traditionally demographic explanation; it could be that high levels of infant and child mortality are behind this trend. We do not have a thorough qualitative understanding of precisely what women mean when they answer questions about their retrospective fertility ideals. It is often assumed that women are reporting their ideal number of surviving children, but it could be that they are reporting their ideal number of children ever born and thereby taking into account the mortality levels that they have experienced. What we need is a thorough qualitative understanding of how women in different contexts understand and answer these questions. A deeper qualitative understanding of the consequences of underachieving fertility ideals in low and middle income countries is also needed. As Casterline and Han point out, the goal of reducing unintended fertility (overachieving fertility ideals) is seen as advancing individual and national agendas, whereas to reduce underachieving of fertility ideals will merely advance individual goals [7]. Despite the long term focus on a rights based approach to family planning, it may be that commitment to allowing individuals to achieve their fertility intentions is limited when those intentions are to have many (more) children [7].

## Supporting information

S1 Table List of Demographic and Health Surveys used in analysis. (XLSX)

S2 Table Percentage of women overachieving, underachieving and equalling fertility desires by educational achievement in 58 Demographic and Health Surveys. (XLSX)
